# Chemical Diversity and Biological Activities of *Phaeosphaeria* Fungi Genus: A Systematic Review

**DOI:** 10.3390/jof4040130

**Published:** 2018-12-05

**Authors:** Amr El-Demerdash

**Affiliations:** 1 Sorbonne Universités, Muséum National d’Histoire Naturelle, Molécules de Communication et Adaptation des Micro-organismes, UMR 7245 CNRS/MNHN, 75005 Paris, France; eldemerdash555@gmail.com; 2 Chemistry Department, Faculty of Science, Mansoura University, Mansoura 35516, Egypt

**Keywords:** *Phaeosphaeria*, pyrazine alkaloids, polyketides, isocoumarins, perylenequinones, anthraquinones, cyclic peptides, bioactivities

## Abstract

Microbial natural products (MNPs) have been identified as important hotspots and effective sources for drug lead discovery. The genus *Phaeosphaeria* (family: Phaeosphaeriaceae, order: Pleosporales), in particular, has produced divergent chemical structures, including pyrazine alkaloids, isocoumarins, perylenequinones, anthraquinones, diterpenes, and cyclic peptides, which display a wide scope of biological potentialities. This contribution comprehensively highlights, over the period 1974–2018, the chemistry and biology of the isolated natural products from the micro-filamentous *Phaeosphaeria* fungi genus. A list of 71 compounds, with structural and biological diversities, were gathered into 5 main groups.

## 1. Introduction

Natural products are a vast and renewable source for novel medicinal products [1,2,3,4,5]. Since the discovery of penicillin in 1928 and streptomycin in 1943, microbial natural products (MNPs) have emerged and have been identified as one of the most powerful prolific sources for drug lead discovery over the past seven decades. Natural product-derived compounds provided impressive and continuous pools for medicinal chemistry applications, and encouraged most of the leading pharmaceutical companies in screening microbial natural extracts for the development of high-throughput libraries [6,7,8]. Recently, the Food and Drug Administration (FDA) declared that natural products and their derivatives represent 38% of all new molecular entities, with 25% coming from microbes, which implies the vital role of microorganisms as a sustainable pipeline in the production of bioactives [7]. Moreover, microbes present a diverse underdeveloped source that extended beyond the terrestrial system to the marine phoma, featuring unusual modes of habitation, including variation in temperature, pressure, acidity, or basicity, which finally affect the structural novelty and complexity. To date, two successful marine microbial natural and synthetic products have been promoted by Nereus Pharmaceuticals in advanced clinical trials for cancer treatment, including plinabulin (phase II), which is a fully synthetic analogue base on the natural diketopeprazine alkaloid halimide, isolated from a marine fungus *Aspergillus* sp., and marizomib “salinosporamide A” (phase I), isolated from the marine actinomycete *Salinispora tropica* [2]. *Phaeosphaeria* is a genus of micro-filamentous fungi belonging to the family Phaeosphaeriaceae (order: Pleosporales), a member of Dothideomycetes, the largest fungal taxon. Most of the *Phaeosphaeria* species are plant pathogens for weeds and grasses. They cause serious infectious, in particular for many important crops plant families like wheat and maize [9]. Early genomic studies were centered only on one species, *Phaeosphaeria nodorum.* These studies disclosed the presence of 48 biosynthetic gene clusters, including 23 PKS, 14 NPRS, four TS, and five PT. Such a high number of gene clusters implies the high capacity of *Phaeosphaeria nodorum* as a producing pool for secondary metabolites. However, at present, only two biosynthetic gene clusters were connected to their metabolites, including SN477 (for isocoumarins-mellein) and SnPKS19 (for alternariol) [10,11,12,13]. The Mycobank databases revealed the presence of 208 recorded *Phaeosphaeria* species, from both terrestrial and marine systems [14]. *Phaeosphaeria* species have produced a diversity of chemical constituents with a wide scope of biological potentialities including cytotoxicity, antimicrobial, anti-tuberculosis, and antibiotic. To the best of our knowledge, the previous chemical investigations were centered only on five species, including *Phaeosphaeria nodorum* (*Septoria nodorum* or *Stagonospora nodorum*)*, Phaeosphaeria* sp., *Phaeosphaeria spartinae, Phaeosphaeria rousseliana*, and *Phaeosphaeria avenaria.* In this communication, we aim to gain the attention of the readers by covering extensively, over the period 1974–2018, the chemical and biological landmarks centered on the microbial natural compounds isolated from the *Phaeosphaeria* fungi genus (Table 1). Attributively, it is clear that *Phaeosphaeria* is a significantly rich source for structurally diverse natural compounds, which exhibit a plethora of bioactivities.

## 2. Chemistry and Biology of Microbial Natural Products Isolated from the Genus *Phaeosphaeria*

In this review, we aim to comprehensively document the chemical and biological aspects of the fungal metabolites exclusively isolated from the *Phaeosphaeria* fungi genus. The isolated compounds are classified into 5 main groups (based on their carbon skeleton) for convenience of handling, and their biological potentialities are enclosed whenever available.

### 2.1. Cyclohexanoids, Naphthoquinones, Anthraquinones, and Phenalenones

**Figure 1:**
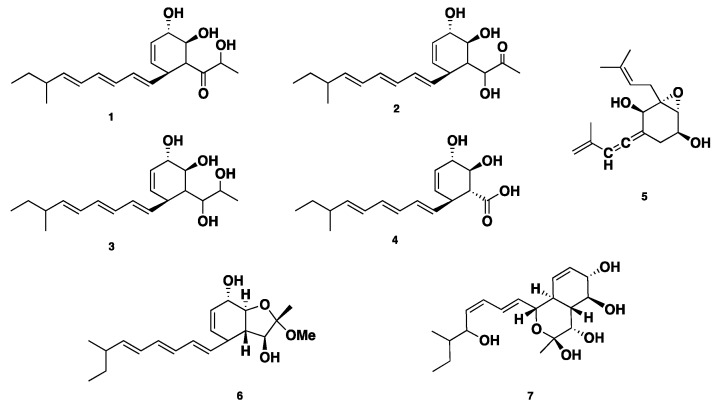
Chemical structures of **1**–**7**.

Four polyketide derived compounds named spartinol A–D (**1**–**4**) were isolated from the endophyte fungus *Phaeosphaeria spartinae*, derived from marine-algae *Ceramium* sp. Spartinol C (**3**) displayed cytotoxicity against human leukocyte elastase (HLE), with an IC_50_ value of 17.7 ± 2.48 μg/mL. Furthermore, compounds **1**–**3** showed no significant cytotoxicity against a set of 36 cancer cell lines at concentrations of 1 μg/mL and 10 μg/mL [15]. Spartinoxide (**5**), an antitumor polyketide-derived cyclohexanoide (featuring an epoxide moiety), was isolated from the same fungus. Compound **5** is an enantiomer of the known compound A82775C. Compound **5** showed potent inhibition of HLE with an IC_50_ value of 1.71 ± 0.30 μg/mL (6.5 μM) [16]. Reinvestigation of the same fungal strain led to the isolation of two further unprecedented bicyclic polyketides, furanospartinol (**6**) and pyranospartinol (**7**). Compounds **6**–**7** showed no remarkable antimicrobial or cytotoxic activities [17] (Figure 1).

**Figure 2:**
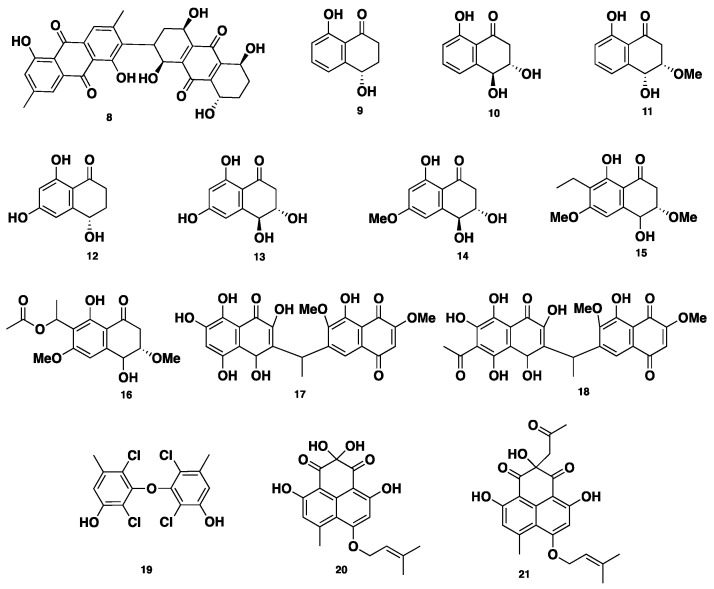
Chemical structures of **8**–**21**.

Phaeosphenone (8), a dimeric anthraquinone compound, was isolated from *Phaeosphaeria* sp. Compound 8 inhibited the growth of wild-type of the Gram-positive bacterial strains *Staphylococcus aureus*, with an MIC of 32–64 µg/mL and an MIC_80_ of 16–32 µg/mL. Moreover, compound 8 showed similar antibacterial activity against three Gram-positive strains, including *Streptococcus pneumoniae*, *Enterococcus faecalis*, and *Bacillus subtilis*, with MICs of 64, 16–32, and 6–32 µg/mL, respectively. Furthermore, compound 8 inhibited the growth of *S. pneumoniae*, with an MIC of 8 µg/mL, when *S. pneumoniae* was cultivated in isosensitet medium. On the other hand, compound 8 showed no activity against two Gram-negative strains, *Haemophilus influenza* and *Escherichia coli*, whereas it was slightly active against *Candida albicans*, with an MIC of 8 µg/mL, indicating a selectivity for Gram-positive organisms. Additionally, compound 8 displayed inhibition of *Staphylococcus aureus* RNA synthesis, with an IC_50_ of 6 µg/mL. Such desired inhibition of RNA synthesis over the protein synthesis was not clear and showed that compound 8 possesses another unknown mechanism of action [18]. Eleven antimycobacterial metabolites, of which eight are naphthalenone/naphthaquinone derivatives, including regiolone (**9**), trihydroxydihydronaphthalenone (**10**), dihydroxymethoxydihydronaphthalenone (**11**), trihydroxydihydronaphthalenone (**12**), 4-hydroxyscytalone (**13**), and trihydroxydimethoxynaphthaquinone (**14**), ethylhydroxyldimethoxy naphthaquinone (**15**), acetylhydroxy-dimethoxy naphthaquinone (**16**), two unsymmetrical naphthoquinone dimers, deacetylkirschsteinin (**17**) and kirschsteinin (**18**), along with a chlorinated diphenyl ether, oxybis (2,4-dichloro-5-methylphenol) (**19**), were isolated from *Phaeosphaeria* sp. BCC8292 (Figure 2). Compounds **9**–**19** were evaluated for their antibacterial activity against *Mycobacterium tuberculosis* and cytotoxicity against several cancer cell lines, including KB, BCA, NCI-H187, and Vero cells. Compounds **11**–**12** displayed significant antibacterial activity, with an MIC of 12.50 µg/mL for each. With respect to their cytotoxicity against BCA cell lines, **12** was found to be more potent with an IC_50_ of 2.96 µg/mL, while **13** was less active with an IC_50_ of 19.16 µg/mL. Compound **15** displayed moderate anti-tuberculosis (TB) activity, with an IC_50_ of 12.50 µg/mL. However, compound **16** demonstrated potent anti-TB activity, with an IC_50_ of 0.39 µg/mL. Meanwhile, compound **16** displayed significant cytotoxicity against KB, NCI-H187, and Vero cell lines, with IC_50_ values of 0.028, 0.25, and 0.33 µg/mL, respectively. Compound **17** showed moderate anti-TB activity, with an MIC of 6.25 µg/mL, however its acetyl congener **18** was not active. None of compounds **9**–**14** and **17**–**18** were active against KB cell lines. Compound **19** displayed weak anti-TB activity, with an MIC of 50 µg/mL, and moderate cytotoxicity against Vero cells, with an IC_50_ of 27.28 µg/mL; however, it was found to be inactive against BCA, KB, and NCI-H187 [19]. Rousselianone A (**20**), a phenalenone-related metabolite, was isolated from *Phaeosphaeria rousseliana.* Compound **20** bears a germinal-gylcol at C-6, and a non-cyclized isoprene moiety, which is not a common structural feature within other phenalenones. Its acetone adduct rousselianone A’ (**21**) was obtained when compound **20** was dissolved in acetone, in the presence of a little acetic acid. Compound **20** displayed, in vivo, a wide significant antifungal activity against five plant pathogens: *Pyricularia oryzae*, *Rhizoctonia solani*, *Puccinia recondita*, *Botrytis cinerea*, and *Phytophthora infestans*. On the contrary, compound **21** showed no antifungal activity [20].

**Figure 3:**
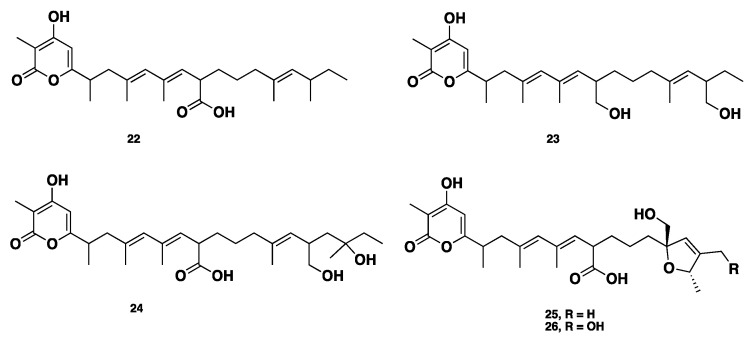
Chemical structures of **22**–**26.**

Recently, alternapyrones B–F (**22**–**26**) (Figure 3), 5 new α-pyrone polyketides, were isolated from the wheat plant pathogen *Parastagonospora nodorum* using a chemical ecogenomics-guided approach. These compounds displayed various bioactivities, including antibacterial, antifungal, antiparasitic, antitumor, and antigermination activities. Alternapyrone F (**26**) was the most active compound, and showed complete inhibition for wheat germination at 100 μg/mL. Although compounds **25**–**26** exhibited potent antigermination activity on wheat seeds, they displayed no remarkable cytotoxicity against tested cancer cell lines. This observation highlighted that the mode of action of cytotoxicity and phytotoxicity (antigermination) is totally different [21].

### 2.2. Isocoumarins, Isobenzofuran, and Related Metabolites

**Figure 4:**
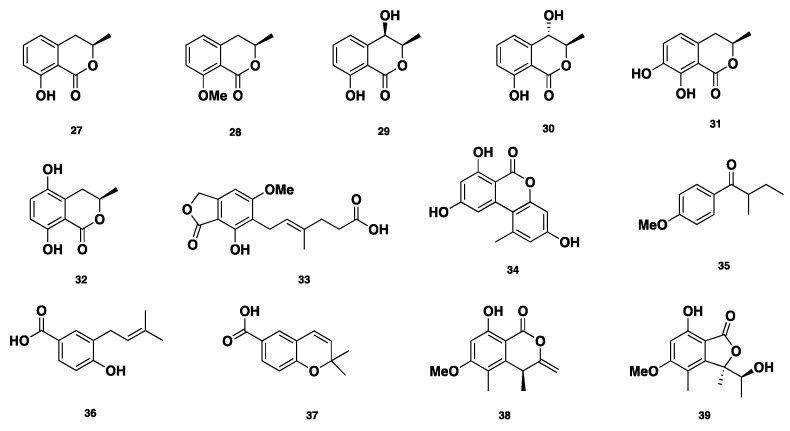
Chemical structures of **27**–**39.**

Six isocoumarin mellein-related compounds (Figure 4), including mellein (**27**), 8-*O*-methylmellein (**28**), (−)-(3*R*,4*R*)-4-hydroxymellein (**29**), (−)-(3*R*,4*S*)-4-hydroxymellein (**30**), 7-hydroxymellein (**31**), and 5-hydroxymellein (**32**), in addition to mycophenolic acid (**33**), alternariol (**34**), and 4-methoxy-(2*S*)-methylbutrophenone (**35**) were isolated from *Phaeosphaeria nodourm*, which is also identified as *Septoria nodorum* or *Stagonospora nodorum* [22,23,24,25,26,27,28,29]. 4-hydroxy-3-prenyl-benzoic acid (**36**) and anofinic acid (**37**) were isolated from *Phaeosphaeria spartinae* [16]. Additionally, (*R*)-4,8-dihydroxy-6-methoxy-4,5-dimethyl-3-methyleneisochroman-1-one (**38**) and (*R*)-7-hydroxy-3-((*S*)-1-hydroxyethyl)-5-methoxy-3,4-dimethylisobenzofuran-1(3H)-one (**39**) were isolated from a Dothideomycete that was identified as *Phaeosphaeria* sp. [30]. Compound **38** showed inhibition against *C. albicans* (DAY185), with an MIC of 86 ± 3 µg/mL. Compound **39** showed mild antimycobacterial activity, with an MIC of 200 µg/mL [31]. Moreover, compounds **35** and **39** displayed antifungal activity against *Cochliobolus miyabanus*, with IC_50_ values of 10 and 0.5 µg/mL, respectively [32].

### 2.3. Perylenequinones

**Figure 5:**
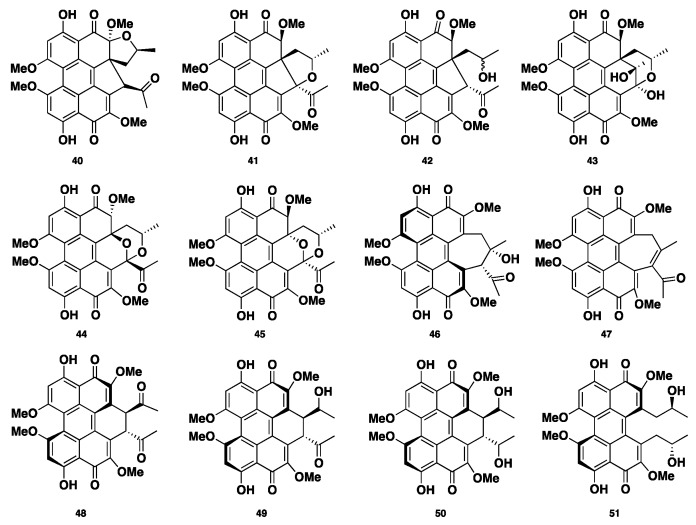
Chemical structures of **40**–**51**.

Phaeosphaerins A–F (**40**–**45**), hypocrellins A and C (**46**–**47**), elsinochromes A–C (**48**–**50**), and (+)-calphostin D (**51**) (Figure 5) were isolated from the endolichenic fungus *Phaeosphaeria* sp. Compounds **40**–**45** showed significant cytotoxicity against the PC3 human prostate cancer cell line, DU145, and LNCaP. Compound **46** was the most active, with IC_50_ values of 2.42 ± 0.13, 9.54 ± 0.27, and 2.67 ± 0.27 µM, respectively. Moreover, compounds **42** and **46** displayed phototoxic activity against human K562, where compound **46** was the most active with an IC_50_ of 0.55 ± 0.03 µM in light, and an IC_50_ of 7.47 ± 0.37 µM in the absence of light [33].

### 2.4. Terpenoide/Steroidal Compounds

**Figure 6:**
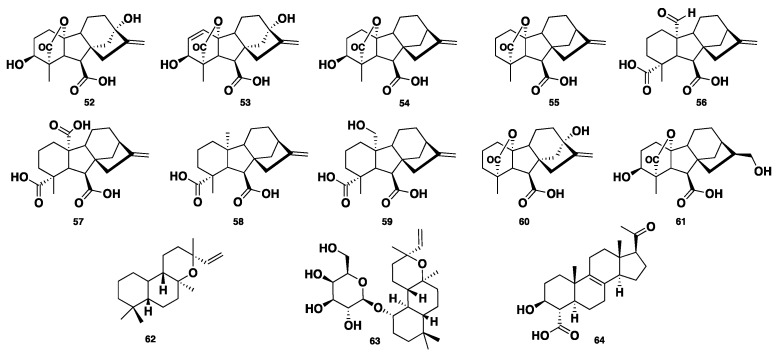
Chemical structures of **52**–**64.**

Gibberellins (GAs) are a distinct group of widely distributed plant-derived diterpenoid metabolites, bearing a common 6/5/6/5 tetracyclic ring system. Essentially, they are plant hormones responsible for the regulation of growth and they also affect other biological processes like metabolism, flowering, germination, and sexual expression. They are mostly isolated from higher plants, algae, and fungi [34]. Six GA metabolites, GA_1_, GA_3_, GA_4_, GA_9_, GA_24_, and GA_25_ (**52**–**57**), were isolated from a *Phaeosphaeria* sp. L487 [35,36,37,38]. Compounds **52**–**53** displayed elongation activity on Chinese cabbage seedlings at concentrations > 0.3 and > 0.1 µg/mL, respectively. Compounds **54**–**55** showed significant inhibition of the growth of Chinese cabbage seedlings at a concentration less than 0.01 µg/mL, whereas compounds **56**–**57** exhibited such activity with MICs of 0.3 and 4 µg/mL. A further four GA congeners, GA_12_, GA_15_, GA_20_, and GA_82_ (**58**–**61**), were identified from *Phaeosphaeria* sp. L487 [39,40]. Two additional gibberellin-related diterpenes, *ent*-13-*epi*-manoyl oxide (**62**) and its 1-galactoside congener, phaeoside (**63**), were isolated from *Phaeosphaeria* sp. L487. Compound **63** was reported as the first fungal diterpene galactoside [41]. Spartopregnenolone (**64**) was isolated from *Phaeosphaeria spartinae.* Compound **64** bears triterpene/steroid structural features. It processes an *endo*-cyclic double bond C8=C9, like lanosterols. Also, the carboxylic group at C-4 is distinct for intermediates between triterpenes and steroids, where the acetyl side chain is typically like that of pregnanes [42] (Figure 6).

### 2.5. Nitrogen-Containing Compounds

**Figure 7:**
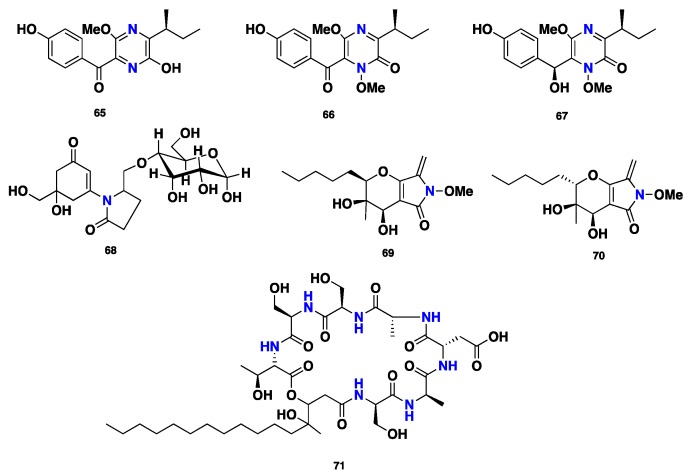
Chemical structures of **65**–**71.**

Three highly substituted pyrazine-containing phytotoxins, (+)-septorine (**65**), *N*-methoxysptorine (**66**), and *N*-methoxyseptorinol (**67**), were isolated from the fungus *Septoria (Phaeosphaeria) nodorum* [43,44,45,46]. Compound **65** displayed inhibition of the growth of wheat *coleoptile mitochondria* [47]. Further 9-*O*-glucosyl mycosporin-2 (**68**) was reported from *Septoria nodorum* [48]. Phaeosphaerides A–B (**69**–**70**), two bicyclic distereoisomers featuring α,β-unsaturated *ene*-amide γ-lactam, were isolated from the endophytic fungus *Phaeosphaeria avenaria*. Compound **69** showed inhibition of STAT-3 and STAT-3-dependent U266 multiple myeloma cells, with IC_50_ values of 0.61 and 6.7 µM, respectively. Additionally, compound **69** exhibited slight cytotoxic activity against the signal transducer and activator of transcription 1 (STAT-1) from U937 cell lines. However, it displayed no activity against STAT-5 from Nb2 cells. Moreover, compound **69** was active against STAT-3 from HepG2 cancer cell lines; however, its diastereomer, phaeosphaeride B (**70**), was inactive [49]. A depsipeptide, phaeofungin (**71**) was isolated from *Phaeosphaeria* sp. Compound **71** displayed moderate antifungal activity against *Candida albicans* and *Candida lusitaniae*, with MIC values of 16–32 and 32 µg/mL, respectively. However, it was slightly more active against *Aspergillus fumigatus* and *Trichophyton mentagrophytes*, with MIC values of 8–16 and 4 µg/mL, respectively. Furthermore, compound **71** exhibited no activity against *Staphylococcus aureus*, even at the high concentration of 32 µg/mL [50] (Figure 7).

## 3. Conclusions and Perspective

A diversity of 71 microbial natural products have been documented. This aforementioned chemical diversity demonstrates that the *Phaeosphaeria* genus is a rich and promising source for structurally divergent secondary metabolites, with a wide scope of bioactivities. However, the number of isolated compounds, compared to the number of *Phaeosphaeria* species, implies that it is still an under-investigated research area, worthy of more chemical and pharmacological explorations by natural product scientists.

## Figures and Tables

**Table 1:** Summary of the natural products isolated from the genus *Phaeosphaeria* and their bioactivities.

Name	Class	Species	Biological Activity	Ref.
Spartinol A–D (**1**–**4**)	Polyketide	*P. spartinae*	Cytotoxic	[15]
Spartinoxide (**5**)	Polyketide	*P. spartinae*	Cytotoxic	[16]
Furanospartinol (**6**)	Polyketide	*P. spartinae*	Antimicrobial, cytotoxic	[17]
Pyranospartinol (**7**)	Polyketide	*P. spartinae*	Antimicrobial, cytotoxic	[17]
Phaeosphenone (**8**)	Polyketide	*Phaeosphaeria* sp.	Antifungal, antibacterial	[18]
**9**–**19**	Polyketide	*Phaeosphaeria* sp.	Cytotoxic, anti-tuberculosis	[19]
Rousselianone A (**20**)	Polyketide	*P. rousseliana*	Antibiotic	[20]
Rousselianone A’ (**21**)	Polyketide	*P. rousseliana*	No activity	[20]
Alternapyrones B–F (**22**–**26**)	Polyketide	*P. nodorum*	Cytotoxic, herbicidal	[21]
**27**–**35**	Polyketide	*P. nodorum*	No activity	[22,23,24,25,26,27,28,29]
**36**–**37**	Polyketide	*P. nodorum*	No activity	[16]
**38**–**39**	Polyketide	*Phaeosphaeria* sp.	Antifungal	[30,31,32]
**40**–**51**	Polyketide	*Phaeosphaeria* sp.	Cytotoxic	[33]
**52**–**63**	Diterpene	*Phaeosphaeria* sp.	Antimicrobial	[34,35,36,37,38,39,40,41]
Spartopregnenolone (**64**)	Steroid	*P. spartinae*	No activity	[42]
**65**–**67**	Pyrazine alkaloid	*P. nodorum*	Antimicrobial	[43,44,45,46,47]
**68**	Pyrrolidone	*P. nodorum*	Phytotoxic	[48]
**69**–**70**	Pyrrolidone	*P. avenaria*	Antifungal, antibacterial	[49]
Phaeofungin (**71**)	Cyclic depsipeptide	*Phaeosphaeria* sp.	Antifungal, antibacterial	[50]

## Abbreviations

IC_50_	Half Maximal Inhibitory Concentration
EC_50_	Half Maximal Effective Concentration
MIC	Minimum Inhibitory Concentration
MIC_80_	Minimum Concentration required to Inhibit 80%
STAT	Signal Transducer and Activator of Transcription
PKS	Polyketide Synthases
NPRS	Non-Ribosomal Peptide Synthetases

## References

[1] Lam K.S. (2007). New aspects of natural products in drug discovery. Trends Microbiol..

[2] Mayer A.M.S., Glaser K.B., Cuevas C., Jacobs R.S., Kem W., Little R.D., McIntosh J.M., Newman D.J., Potts B.C., Shuster D.E. (2010). The odyssey of marine pharmaceuticals: A current pipeline perspective. Trends Pharmacol. Sci..

[3] Cragg G.M., Newman D.J. (2013). Natural products: A continuing source of novel drug leads. Biochem. Biophys. Acta.

[4] Shen B. (2015). A new golden age of natural products drug discovery. Cell.

[5] Patridge E., Gareiss P., Kinch M.S., Hoyer D. (2016). An analysis of FAD-approved drugs: Natural products and their derivatives. Drug Discov. Today.

[6] Hung T., Lin S. (2017). Microbial natural products: A promising source for drug discovery. J. Appl. Microbiol. Biochem..

[7] Newman D.J., Cragg G.M. (2016). Natural products as sources of new drugs from 1981 to 2014. J. Nat. Prod..

[8] Harvey A.L., Edrada-Ebel R., Quinn R.J. (2015). The re-emergency of natural products for drug discovery in the genomics era. Nat. Rev. Drug. Discov..

[9] Shoemaker R.A., Babcock C.E. (1989). Phaeosphaeria. Can. J. Bot..

[10] Hane J.K., Lowe R.G.T., Solomon P.S., Tan K.C., Schoch C.L., Spatafora J.W., Crous P.W., Kodira C., Birren B.W., Galagan J.E. (2007). Dothideomycete plant interactions illuminated by genome sequencing and EST analysis of the wheat pathogen *Stagonospora nodorum*. Plant Cell.

[11] Chooi Y.H., Muria-Gonzalez M.J., Solomon P.S. (2014). A genome wide survey of the secondary metabolite biosynthesis genes in the wheat pathogen *Parastagonospora nodorum*. Mycology.

[12] Chooi Y.H., Krill C., Barrow R.A., Chen S., Trengove R., Oliver R.P., Solomon P.S. (2015). An In planta expressed polyketide synthase produces (*R*)-mellein in the wheat pathogen *Parastagonospora nodorum*. Appl. Environ. Microbiol..

[13] Chooi Y.H., Muria-Gonzalez M.J., Mead O.L., Solomon P.S. (2015). SnPKS19 encodes the polyketide synthase for alternariol mycotoxin biosynthesis in the wheat pathogen *Parastagonospora nodorum*. Appl. Environ. Microbiol..

[14] Mycobank: Phaeosphaeria. http://www.mycobank.org/Biolomics.aspx?Table=Mycobank&Rec=37177&Fields=All.

[15] Elsebaia M.F., Kehrausa S., Guütschowb M., Königa G.M. (2009). New polyketides from the marine-derived fungus *Phaeosphaeria spartinae*. Nat. Prod. Commun..

[16] Elsebaia M.F., Kehrausa S., Guetschow M., Königa G.M. (2010). Spartinoxide, a new enantiomer of A82775C with inhibitory activity toward HLE from the marine-derived fungus *Phaeosphaeria spartinae*. Nat. Prod. Commun..

[17] Elsebaia M.F., El Maddah F., Kehrausa S., Königa G.M. (2015). New Bicyclo-spartinols from the Marine-derived Fungus *Phaeosphaeria spartinae*. Nat. Prod. Commun..

[18] Zhang C., Ondeyka J.G., Zink D.L., Basilio A., Vicente F., Collado J., Platas G., Bills G., Huber J., Dorso K. (2008). Isolation, structure, and antibacterial activity of Phaeosphenone from a *Phaeosphaeria* sp. discovered by antisense strategy. J. Nat. Prod..

[19] Pittayakhajonwut P., Sohsomboon P., Dramae A., Suvannakad R., Lapanun S., Tantichareon M. (2008). Antimycobacterial substances from *Phaeosphaeria* sp. BCC8292. Planta Med..

[20] Xiao J.Z., Kumazawa S., Tomita H., Yoshikawa N., Kimura C., Mikawa T., Rousselianone A. (1993). novel antibiotic related to phenalenone produced by *phaeosphaeria rousseliana*. J. Antibiot..

[21] Li H., Hu J., Wei H., Solomon P.S., Vuong D., Lacey E., Stubbs K.A., Piggott A.M., Chooi Y.-H. (2018). Chemical Ecogenomics-Guided Discovery of Phytotoxic α-Pyrones from the Fungal Wheat Pathogen *Parastagonospora nodorum*. Org. Lett..

[22] Bousquet J.F., Skajennikoff M. (1974). Isolation and mode of action of a phytotoxin produced by *Septoria nodorum*. Phytopathol. Z..

[23] Devys P.M., Bousquet J.F., Skajennikoff M., Barbier M. (1974). Ľochracine (meiléine), phytotoxine isolée du milieu de culture de *Septoria nodorum* Berk. Phytopathol. Z..

[24] Devys M., Bousquet J.F., Kollmann A., Barbier M. (1980). Dihydroisocoumarines et acide mycophénolique du milieu de culture du champignon phytopathogéne *Septoria nodorum*. Phytochemistry.

[25] Devys M., Barbier M. (1992). Isolation of new (−)-(3*R*,4*S*)-4-hydroxymellein from the fungus *Septoria nodorum* Berk. Z. Naturforsch..

[26] Devys M., Barbier M., Bousquet J.F., Kollmann A. (1994). Isolation of the (−)-(3*R*)-5-hydroxymellein from the fungus *Septoria nodorum*. Phytochemistry.

[27] Bousquet J.F., Kollmann A. (1998). Variation in metabolite production by *Septoria nodorum* isolates adapted to wheat or to barley. J. Phytopathol..

[28] Tan K.-C., Trengove R., Maker G., Oliver R., Solomon P. (2009). Metabolite profiling identifies the mycotoxin alternariol in the pathogen *Stagonospora nodorum*. Metabolomics.

[29] Yang X.-L., Awakawa T., Wakimoto T., Abe I. (2013). Induced biosyntheses of a novel butyrophenone and two aromatic polyketides in the plant pathogen *Stagonospora nodorum*. Nat. Prod. Bioprospect..

[30] Gerea A.L., Branscum K.M., King J.B., You J., Powell D.R., Miller A.N., Spear J.R., Cichewicz R.H. (2012). Secondary metabolites produced by fungi derived from a microbial mat encountered in an iron-rich natural spring. Tetrahedron Lett..

[31] Chinworrungsee M., Kittakoop P., Isaka M., Chanphen R., Tanticharoen M., Thebtaranonth Y. (2002). Halorosellins A and B, unique isocoumarin glucosides from the marine fungus *Halorosellinia oceanica*. J. Chem. Soc. Perkin Trans..

[32] Tayone W.C., Honma M., Kanamaru S., Noguchi S., Tanaka K., Nehira T., Hashimoto M. (2011). Stereochemical investigations of isochromenones and isobenzofuranones isolated from *Leptosphaeria* sp. KTC 727. J. Nat. Prod..

[33] Li G., Wang H., Zhu R., Sun L., Wang L., Li M., Li Y., Liu Y., Zhao Z., Lou H. (2012). Phaeosphaerins A-F, cytotoxic perylenequinones from an endolichenic fungus, *Phaeosphaeria* sp.. J. Nat. Prod..

[34] Stowe B.B., Yamaki T. (1957). the history and physiological action of the gibberellins. Ann. Rev. Plant Physiol..

[35] Sassa T., Suzuki K., Haruki E. (1989). Isolation and identification of gibberellins A4 and A9 from a fungus *Phaeosphaeria* sp.. Agric. Biol. Chem..

[36] Sassa T., Suzuki K. (1990). Metabolism of gibberellin A_9_ to gibberellin A_4_ n a new gibberellin producing fungus *Phaeosphaeria* sp.. Agric. Biol. Chem..

[37] Kawaide H., Sassa T. (1993). Accumulation of gibberellin A1 and the metabolism of gibberellin A9 to gibberellin A1 in a *Phaeosphaeria* sp. L487 Culture. Biosci. Biotechnol. Biochem..

[38] Sassa T., Kawaide H., Takarada T. (1994). Identification of gibberellins A4, A9, and A24 from *Phaeosphaeria* sp. L487 cultured in a chemically defined medium. Biosci. Biotechnol. Biochem..

[39] Kawaide H., Sassa T., Kamiya Y. (1995). Plant-like biosynthesis of gibberellin A1 in the fungus *Phaeosphaeria* sp. L487. Phytochemistry.

[40] Seto H., Sassa T., Kawaide H., Shigihara T., Uzawa J., Yoshida S. (1995). Isolation and stereo controlled synthesis of a 17-hydroxy-16β,17-dihydrogibberellin, GA82. Tetrahedron Lett..

[41] Kenmoku H., Sugai T., Yajima H., Sassa T. (2004). Phaeoside, a novel galactoside of hydroxymanoyl oxide from the gibberellin A1-producing *Phaeosphaeria* sp. L487. Biosci. Biotechnol. Biochem..

[42] Elsebai M.F., Kehraus S., König G.M. (2013). Caught between triterpene- and steroid-metabolism: 4α-Carboxylic pregnane-derivative from the marine alga-derived fungus *Phaeosphaeria spartinae*. Steroids.

[43] Devys M., Bousquet J.F., Kollmann A., Barbier M. (1978). La septorine, nouvelle pyrazine substituée du milieu de culture de *Septoria nodorum* Berk., champignon phytopathogéne. CR Acad. Sci..

[44] Devys M., Barbier M., Kollmann A., Bousquet J.F. (1982). Septorine and *N*-methoxyseptorine substituted pyrazines from the fungus *Septoria nodorum* Berk. Tetrahedron Lett..

[45] Devys M., Barbier M., Kollmann A., Bousquet J.F. (1992). *N*-methoxyseptorinol a substituted pyrazine from the fungus *Septoria nodorum*. Phytochemistry.

[46] Barbier M., Devys M., Bousquet J.F., Kollmann A. (1994). Absoulte stereochemistry of *N*-methoxyseptorinol isolated from the fungus *Septoria nodorum*. Phytochemistry.

[47] Bousquet J.F., Belhomme de Franqueville H., Kollmann A., Fritz R. (1980). Action de la septorine, phytotoxine synthetisee par *Septoria nodorum*, sur la phosphorylation oxydative dans les mitochondries isolees de Coleoptiles de Ble. Can. J. Bat..

[48] Bouillant M.L., Pittet J.L., Bernillon J., Favre-Bonvin J., Arpin N. (1982). Mycosporins from *Ascochyta pisi*, *Cladosporium herbarum* and *Septoria nodorum*. Phytochemistry.

[49] Maloney K.N., Hao W., Xu J., Gibbons J., Hucul J., Roll D., Brady S.F., Schroeder F.C., Clardy J. (2006). Phaeosphaeride A, an Inhibitor of STAT3-dependent signaling isolated from an endophytic fungus. Org. Lett..

[50] Singh S.B., Ondeyka J., Harris G., Herath K., Zink D., Vicente F., Bills G., Collado J., Platas G., Ganzalez del Val A. (2013). Isolation, Structure, and Biological Activity of Phaeofungin, a cyclic lipodepsipeptide from a *Phaeosphaeria* sp. using the genome-wide *Candida albicans* fitness test. J. Nat. Prod..

